# The incidence and determinants of traumatic brain injury deaths occurring outside hospital in Australia

**DOI:** 10.1111/1742-6723.70051

**Published:** 2025-05-02

**Authors:** Gerard M O'Reilly, Afsana Afroz, Kate Curtis, Biswadev Mitra, Yesul Kim, Emma Solly, Courtney Ryder, Kate Hunter, Delia V Hendrie, Nick Rushworth, Jin Tee, Mark C Fitzgerald

**Affiliations:** ^1^ National Trauma Research Institute Alfred Health Melbourne Victoria Australia; ^2^ Emergency and Trauma Centre Alfred Health Melbourne Victoria Australia; ^3^ School of Public Health and Preventive Medicine Monash University Melbourne Victoria Australia; ^4^ Susan Wakil School of Nursing and Midwifery, Faculty of Medicine and Health University of Sydney Sydney New South Wales Australia; ^5^ Emergency Department Wollongong Hospital, Illawarra Shoalhaven Local Health District Wollongong New South Wales Australia; ^6^ The George Institute for Global Health UNSW Sydney New South Wales Australia; ^7^ School of Translational Medicine Monash University Melbourne Victoria Australia; ^8^ College of Medicine and Public Health Flinders University Adelaide South Australia Australia; ^9^ School of Population Health Curtin University Perth Western Australia Australia; ^10^ Brain Injury Australia Sydney New South Wales Australia; ^11^ Neurosurgery Alfred Health Melbourne Victoria Australia; ^12^ Trauma Service Alfred Health Melbourne Victoria Australia

**Keywords:** coronial data, injury, prehospital care, trauma, traumatic brain injury

## Abstract

**Objective:**

To identify the determinants of death occurring outside of hospital following moderate to severe traumatic brain injury (msTBI) across Australia.

**Methods:**

Design, setting: Retrospective observational study using National Coronial Information System (NCIS) data. Participants: People who died during the five‐year study period between 2015 and 2020 and were recorded in the NCIS as having intracranial injury as a cause or contributor to death. Major outcome measures: The primary outcome was the location of death, specifically whether death occurred outside an acute hospital setting.

**Results:**

There were 3751 deaths with msTBI, of which 1064 (28.4%) occurred outside of an acute hospital setting and 605 (16.1%) occurred outside any medical service. The odds of death occurring outside hospital were lower for male patients (odds ratio [OR]: 0.6, 95% confidence interval [CI]: 0.5–0.7), penetrating injuries (OR 5.2, 95% CI: 3.0–8.9) and highest in the Northern Territory followed by Queensland. The odds of death occurring outside *any* medical service area (e.g. hospital, rehabilitation, nursing home) were higher for: younger adults (OR 3.6, 95% CI: 1.0–12.7), those with penetrating injuries (OR 8.9, 95% CI: 4.5–17.3), and where the time between injury and death was less than 24 h. The odds of death outside any medical service area were less for people with msTBI in South Australia (OR 0.1, 95% CI 0.0–0.2).

**Conclusion:**

Approximately, one in six msTBI deaths occurred outside of any medical service area. Opportunities exist to improve access to emergency care for people sustaining msTBI across Australia.

## Graphical abstract

Distribution of TBI cases by location of death

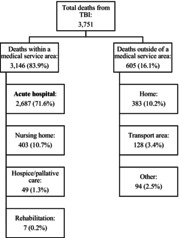

**Key findings**

- More than one quarter of deaths following traumatic brain injury occurred outside an acute hospital setting and approximately one in six deaths occurred outside any medical service area.
- A significant proportion of people who die following traumatic brain injury may not have access to the acute care system.

## Introduction

Among all types of injury, traumatic brain injury (TBI) is the largest contributor to global death and disability and confers a significant socioeconomic and healthcare burden.^1^, ^2^ In Australia, TBI carries an estimated annual cost of $8.6 billion.^3^ Road traffic incidents are the leading cause of TBI worldwide, accounting for approximately 60% of TBI events, followed by falls.^4^ However, in high‐income countries, there has been an epidemiological transition over time, where the proportion of TBIs due to traffic incidents has decreased and the proportion of TBIs due to falls has increased. Consequently, in high‐income countries, falls are the primary cause of TBI.^5^, ^6^, ^7^, ^8^

In Australia, the most recent available research from July 2015 to June 2020 reported that falls are the most common cause of death in hospital following moderate to severe TBI (msTBI) (52.8%) followed by transport incidents (33.8%).^9^ Furthermore, there were 2437 deaths among msTBI patients in major trauma centres across Australia over a recent five‐year period (approximately 487 deaths annually).^9^ However, this figure does not account for deaths occurring outside of hospital or in non‐major trauma hospitals. Further, the incidence of out‐of‐hospital death following msTBI across subgroups defined by age, gender, injury cause, injury location and place of death has not been described.

Dying outside of hospital after TBI, and particularly prior to any hospital care, may, for some patients, be an indication of inadequate access to timely acute trauma care. Understanding the incidence and determinants of death out of hospital following msTBI across Australia may be valuable for effectively identifying and addressing any deficiencies in the existing system‐level strategies for injury prevention and timely emergency care.

The aim of the present study, as part of the Australian TBI National Data (ATBIND) Project, was to address this research gap by analysing data from the NCIS for all Australia. Specifically, the aim was to determine the incidence of death following msTBI and to establish the determinants of out‐of‐hospital death following msTBI.

## Methods

The data for the present study were collected from the National Coronial Information System (NCIS) database. The NCIS is an online database managed by the Victorian Department of Justice and Community Safety that stores and retrieves information about coronial cases in Australia and New Zealand.^10^ It encompasses data on all reported deaths that have been investigated by the coroner since July 2000. The NCIS database includes coded information regarding the nature and cause of the injury event for patients who die following injury. In Australia, deaths from injury are reportable to the coroner.^10^

Cases were included in the present study if they had a medical cause of death (or medical condition contributing to death) due to brain injury as defined by the ICD10 cause of death codes contained under S06 (intracranial injury) and died between 1 July 2015 and 30 June 2020. These dates were chosen to allow comparison with previous work by the ATBIND Project which determined the incidence of msTBI deaths in major trauma hospitals across Australia during the same 5‐year time period.^9^

The primary outcome for the present study was the location of death, namely whether or not the death occurred outside an acute hospital setting. In addition to the incidence of death by location, the determinants of death out of hospital were examined using logistic regression analyses. The exposure variables considered were age, sex, Remote Area Index (RAI), Australian state or territory where the death occurred, primary mechanism of injury and time between injury and death. A secondary analysis was conducted with location of death by ‘Medical Service Area’ or not as the outcome; this was added to account for the potential that some people with msTBI who died in a palliative care or nursing home setting were not necessarily seeking (and therefore failing) to reach an acute care facility. That is, these patients might not necessarily be perceived as having an unwanted delay to acute hospital care. For this secondary analysis, the same set of candidate exposure variables were considered.

Univariable logistic regression analyses were used to test for the crude (unadjusted) association between dying out‐of‐hospital and each of the candidate variables. Then, to determine the independent determinants of death occurring out‐of‐hospital, following msTBI, multivariable logistic regression analyses were conducted. All variables considered in the univariable regression analyses were included in the final regression models.

Symmetrical numerical data have been summarised using mean and standard deviation (SD), skewed numerical data or ordinal data have been summarised using median and interquartile range (IQR) and nominal data have been summarised using frequency and percentage (%). The measure of association reported for the logistic regression analyses was the odds ratio (OR), with 95% confidence intervals (95% CI). A *P*‐value of <0.05 was considered statistically significant. Data were prepared and analysed using STATA, version 17 (College Station, TX, USA) statistical software.

### Ethics statement

Approval for conducting this study was obtained from the Justice Human Health Research Ethics Committee (JHREC) on 1st July 2022 (JHREC reference number: *CF*/22/8319). This process includes a legislated waiver of consent.

## Results

**Figure 1:**
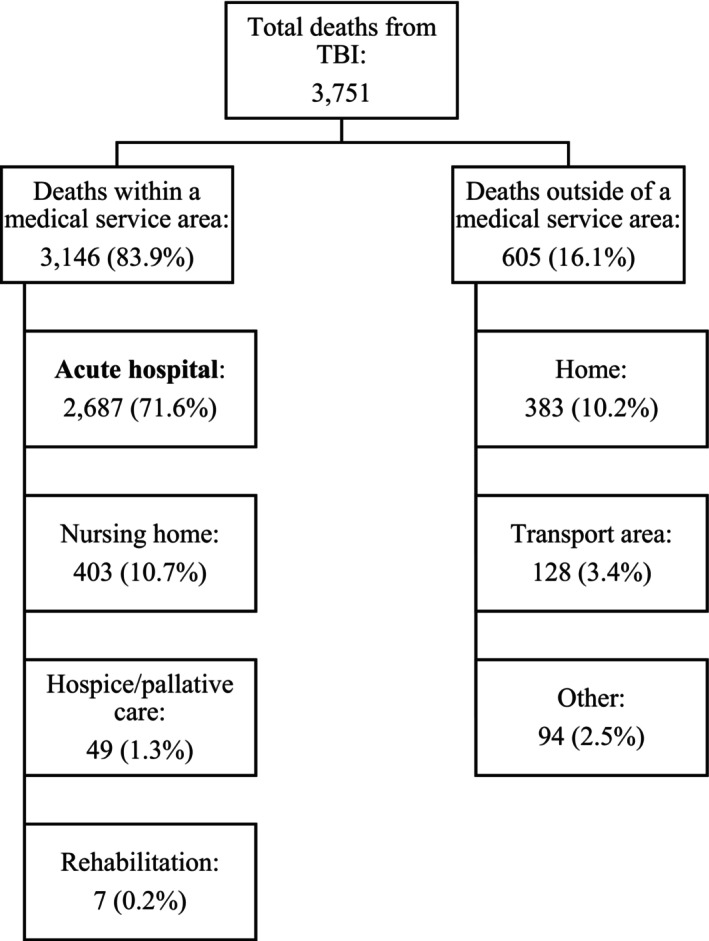
Distribution of cases by location of death.

After removing duplicates, a total of 3751 cases were eligible for analysis, of which 1064 (28.4%) died out of the acute hospital setting. Of those people who died out of acute hospital, 459 (12.2%) died in a healthcare facility not providing acute care (i.e. rehabilitation, hospice, nursing home) with the remaining 605 (16.1%) dying outside any medical service area. Table 1 and Figure 1 provide a breakdown of the participants' locations of death.

**TABLE 1:** Distribution of cases based on location of death

Location	Subgroups	Number	% of total
Medical service area		3146	83.9
	Acute hospital	2687	71.6
	Rehabilitation	7	0.2
	Hospice/Palliative care	49	1.3
	Nursing home	403	10.7
Out of medical service area		605	16.1
	Home	383	10.2
	Transport area†	128	3.4
	Other‡	94	2.5
Total		3751	100

† Transport area – Public highway, freeway, street or road.

‡ Other – Commercial area, countryside, firm, construction area, cultural area, educational area, sports area.

The results of the univariable analyses for any crude or unadjusted association between death out of hospital and the predefined selection of candidate exposure variables are presented in Table 2. Individuals aged 18–64 years were found to have a 2.4‐fold higher odds of out‐of‐acute hospital death (95% CI: 1.2–4.7). Participants residing in inner regional Australia, outer regional Australia, and remote/very remote Australia had higher odds of out‐of‐acute hospital death (OR 1.5; 95% CI: 1.2–1.8; OR 2.2; 95% CI: 1.7–2.8; and OR 1.7, 95% CI: 1.1–2.8; respectively). Additionally, participants who died in Queensland (QLD), Tasmania (TAS), New South Wales (NSW) and the Northern Territory (NT) had a significantly higher odds of death outside of acute hospitals, with ORs ranging from 2.7 to 5.3 compared to participants who died in the Australian Capital Territory (ACT). People who died following penetrating injury had significantly higher odds of their death occurring outside an acute hospital (OR 19.0; 95% CI: 12.9–27.9). Time between incident and death of less than 1 h significantly increased the odds (OR 15.2; 95% CI: 11.8–19.6) of the death occurring out of hospital.

**TABLE 2:** Univariable analysis for association with death outside of an acute hospital

Variable	Missing	Subgroups	Total	Out of hospital	In hospital	OR (95% CI)	*P*‐value
Age in years			3751	1064 (28.4)	2687 (71.6)	0.985 (0.982–0.988)	<0.001
Age groups, *n* (%)		0–17 years	49 (1.3)	12 (1.1)	37 (1.4)	Reference	
		18–64 years	900 (24.0)	395 (37.1)	505 (18.8)	2.4 (1.2–4.7)	0.009
		≥65+ years	2802 (74.7)	657 (61.8)	2145 (79.8)	0.9 (0.5–1.8)	0.864
Sex, *n* (%)		Female	1440 (38.4)	406 (38.2)	1034 (38.5)	Reference	
		Male	2311 (61.6)	658 (61.8)	1653 (61.5)	1.0 (0.9–1.2)	0.854
Residence – Remote Area Index, *n* (%)	535 (14.3)	Major cities in Australia	2195 (58.5)	563 (60.1)	1632 (71.6)	Reference	
		Inner regional Australia	643 (17.1)	215 (23.0)	428 (18.8)	1.5 (1.2–1.8)	<0.001
		Outer regional Australia	306 (8.2)	132 (14.1)	174 (7.6)	2.2 (1.7–2.8)	<0.001
		Remote/very remote Australia	72 (1.9)	27 (2.9)	45 (2.0)	1.7 (1.1–2.8)	0.030
Death – State/Territory, *n* (%)	20 (0.5)	ACT	66 (1.8)	11 (1.0)	55 (2.1)	Reference	
		NSW	399 (10.6)	149 (14.1)	250 (9.4)	3.0 (1.5–5.9)	0.002
		NT	48 (1.3)	17 (1.6)	31 (1.2)	2.7 (1.1–6.6)	0.024
		QLD	231 (6.2)	119 (11.2)	112 (4.2)	5.3 (2.6–10.7)	<0.001
		SA	608 (16.2)	151 (14.3)	457 (17.1)	1.7 (0.8‐3.2)	0.144
		TAS	118 (3.2)	46 (4.3)	72 (2.7)	3.2 (1.5–6.7)	0.002
		VIC	1723 (45.9)	421 (39.7)	1302 (48.8)	1.6 (0.8–3.1)	0.152
		WA	538 (14.3)	146 (13.8)	392 (14.7)	1.9 (0.9–3.7)	0.071
Incident – primary mechanism, *n* (%)		Blunt force	3231 (86.1)	790 (74.3)	2441 (90.8)	Reference	
		Piercing, penetrating force	229 (6.1)	197 (18.5)	32 (1.2)	19.0 (12.9–27.9)	<0.001
		Other†	291 (7.8)	77 (7.2)	214 (8.0)	1.1 (0.8–1.5)	0.446
Time between incident and death	58 (1.6)	>7 days	1071 (29.0)	276 (26.2)	795 (30.1)	Reference	
		24 h to ≤7 days	1429 (38.7)	167 (15.9)	1262 (47.8)	0.4 (0.3–0.5)	<0.001
		1 to ≤24 h	564 (15.3)	80 (7.6)	484 (18.3)	0.5 (0.4–0.6)	<0.001
		0 to ≤1 h	629 (17.3)	529 (50.3)	100 (3.8)	15.2 (11.8–19.6)	<0.001

† Burns and other trauma.

The results of the multivariable logistic regression analysis, to identify exposure variables with an independent association with death outside acute hospitals, are presented in Table 3. The odds of death occurring outside hospital were lower for male patients (OR: 0.6, 95% CI: 0.5–0.7), higher for penetrating injuries (OR 5.2, 95% CI: 3.0–8.9) and highest in TAS followed by NT. The odds of death occurring outside hospital decreased when the time of death was more than 1 h after injury.

**TABLE 3:** Results of multivariable analysis for independent associations with death outside of an acute hospital

Variable	Subgroups	OR (95% CI)	*P*‐value
Age groups	0–17 years	Reference	
	18–64 years	3.0 (1.1–8.4)	0.035
	≥65+ years	2.9 (1.0–8.2)	0.040
Sex	Female	Reference	
	Male	0.6 (0.5–0.7)	<0.001
Residence – Remote Area Index	Major cities in Australia	Reference	
	Inner regional Australia	1.1 (0.8–1.4)	0.600
	Outer regional Australia	1.3 (0.9–1.8)	0.214
	Remote/very remote Australia	0.7 (0.3–1.4)	0.311
Death – State/Territory	ACT	Reference	
	NSW	1.7 (0.7–4.2)	0.243
	NT	4.2 (1.3–13.9)	0.018
	QLD	3.0 (1.2–7.7)	0.019
	SA	2.4 (1.0–5.6)	0.051
	TAS	4.6 (1.7‐12.3)	0.002
	VIC	2.1 (0.9–4.9)	0.090
	WA	2.4 (1.0–5.8)	0.045
Incident – Primary mechanism	Blunt force	Reference	
	Piercing, penetrating force	5.2 (3.0–8.9)	<0.001
	Other†	0.6 (0.4–0.8)	0.004
Time between incident and death	>7 days	Reference	
	24 h to ≤7 days	0.3 (0.2–0.4)	<0.001
	1 to ≤24 h	0.4 (0.3–0.6)	<0.001
	0 to ≤1 h	13.4 (9.7–18.6)	<0.001

† Burns and other trauma.

Table 4 provides the results of any crude or unadjusted association between the death occurring outside of any medical service area (i.e. acute hospital, rehabilitation, hospice, nursing home) and the predefined selection of candidate exposure variables. Individuals aged 18–64 years and males had a 2.3‐fold (95% CI: 1.2–4.4) and 2.1‐fold (95% CI: 1.7–2.5), respectively, higher odds of death occurring out‐of‐medical service area. Participants residing in inner regional Australia, outer regional Australia, and remote/very remote Australia had higher odds of death out‐of‐medical service area (OR 1.6; 95% CI: 1.3–2.0; OR 2.9; 95% CI: 2.2–3.8; and OR 3.3, 95% CI: 2.0–5.5; respectively). Additionally, participants who died in QLD and NSW had a significantly increased odds of their death occurring out‐of‐medical service area, (OR 5.0; 95% CI: 2.5–10.1 and OR 2.8; 95% CI: 1.4–5.6, respectively) compared to people who died in the ACT. People who died following penetrating injury had significantly higher odds of their death occurring outside any medical service area (OR 49.3 95% CI: 33.4–72.7). In contrast, individuals aged 65 years and above (OR 0.2, 95% CI: 0.1–0.5), and those who died in South Australia (SA) (OR 0.2, 95% CI: 0.1–0.5) had lower odds of death out‐of‐medical service area. As the time between injury and death increased, the odds of death occurring out‐of‐medical service area decreased.

**TABLE 4:** Univariable analysis for association with death outside of any medical service area

Variable	Missing	Subgroups	Total	Out of medical service area	In medical service area	OR (95% CI)	*P*‐value
Age in years			3751	605 (16.1)	3146 (83.9)	0.96 (0.95–0.96)	<0.001
Age groups, *n* (%)		0–17 years	49 (1.3)	12 (2.0)	37 (1.2)	Reference	
		18–64 years	900 (24.0)	383 (36.3)	517 (16.4)	2.3 (1.2–4.4)	0.015
		≥ 65+ years	2802 (74.7)	210 (34.7)	2592 (82.4)	0.2 (0.1–0.5)	<0.001
Sex, *n* (%)		Female	1440 (38.4)	151 (25.0)	1289 (41.0)	Reference	
		Male	2311 (61.6)	454 (75.0)	1857 (59.0)	2.1 (1.7–2.5)	<0.001
Residence – Remote Area Index, *n* (%)	535 (14.3)	Major cities in Australia	2195 (58.5)	301 (54.4)	1894 (71.1)	Reference	
		Inner regional Australia	643 (17.1)	131 (23.7)	512 (19.2)	1.6 (1.3–2.0)	<0.001
		Outer regional Australia	306 (8.2)	96 (17.4)	210 (7.9)	2.9 (2.2–3.8)	<0.001
		Remote/very remote Australia	72 (1.9)	25 (4.5)	47 (1.8)	3.3 (2.0–5.5)	<0.001
Death – State/Territory, *n* (%)	20 (0.5)	ACT	66 (1.8)	11 (1.8)	55 (1.8)	Reference	
		NSW	399 (10.6)	144 (23.8)	255 (8.1)	2.8 (1.4–5.6)	0.003
		NT	48 (1.3)	10 (1.7)	38 (1.2)	1.3 (0.5–3.4)	0.572
		QLD	231 (6.2)	116 (19.2)	115 (3.7)	5.0 (2.5–10.1)	<0.001
		SA	608 (16.2)	25 (4.1)	583 (18.6)	0.2 (0.1–0.5)	<0.001
		TAS	118 (3.2)	25 (4.1)	93 (3.0)	1.3 (0.6–2.9)	0.460
		VIC	1723 (45.9)	194 (32.1)	1529 (48.6)	0.6 (0.3–1.2)	0.179
		WA	538 (14.3)	78 (12.9)	460 (14.6)	0.8 (0.4–1.7)	0.639
Incident – Primary mechanism, *n* (%)		Blunt force	3231 (86.1)	359 (59.3)	2872 (91.3)	Reference	
		Piercing, penetrating force	229 (6.1)	197 (32.6)	32 (1.0)	49.3 (33.4–72.7)	<0.001
		Other†	291 (7.8)	49 (8.1)	242 (7.7)	1.6 (1.2–2.2)	0.004
Time between incident and death	58 (1.6)	>7 days	1071 (29.0)	13 (2.2)	1058 (34.2)	Reference	
		24 h to ≤7 days	1429 (38.7)	20 (3.3)	1409 (45.6)	1.2 (0.6–2.3)	<0.687
		1 to ≤24 h	564 (15.3)	63 (10.5)	501 (16.2)	10.2 (5.6–18.8)	<0.001
		0 to ≤1 h	629 (17.0)	504 (84.0)	125 (4.0)	328.1 (183.6–586.6)	<0.001

† Burns and other trauma.

Table 5 displays the results of the multivariable logistic regression analysis, to identify an independent association between death occurring out of a medical service area and the candidate exposure variables. The odds of death occurring outside *any* medical service area (e.g. hospital, rehabilitation, nursing home) were higher for: younger adults (OR 3.6, 95% CI: 1.0–12.7), those with penetrating injuries (OR 8.9, 95% CI: 4.5–17.3), and where the time between injury and death was less than 24 h. The odds of death outside any medical service area were less for people with msTBI in South Australia (OR 0.1, 95% CI 0.0–0.2).

**TABLE 5:** Results of multivariable regression analysis for independent associations with death outside of any medical service area

Variable	Subgroups	OR (95% CI)	*P*‐value
Age groups	0–17 years	Reference	
	18–64 years	3.6 (1.0–12.7)	<0.050
	≥65+ years	1.0 (0.3–3.7)	0.960
Sex	Female	Reference	
	Male	1.0 (0.7–1.5)	0.872
Residence – Remote Area Index	Major cities in Australia	Reference	
	Inner regional Australia	0.6 (0.4–1.0)	0.051
	Outer regional Australia	0.8 (0.5–1.5)	0.530
	Remote/very remote Australia	1.4 (0.5–3.9)	0.474
Death – State/Territory	ACT	Reference	
	NSW	1.0 (0.3–3.3)	0.993
	NT	1.3 (0.2–7.2)	0.784
	QLD	1.8 (0.5–6.3)	0.341
	SA	0.1 (0.0–0.2)	<0.001
	TAS	3.1 (0.7–13.2)	0.134
	VIC	0.7 (0.2–2.3)	0.579
	WA	1.0 (0.3–3.3)	0.960
Incident – Primary mechanism	Blunt force	Reference	
	Piercing, penetrating force	8.9 (4.5–17.3)	<0.001
	Other†	0.3 (0.2–0.6)	<0.001
Time between incident and death	>7 days	Reference	
	24 h to ≤7 days	0.7 (0.3–1.5)	0.372
	1 to ≤24 h	6.2 (3.3–11.8)	<0.001
	0 to ≤1 h	291.2 (149.2–567.4)	<0.001

† Burns and other trauma.

## Discussion

The results of this population‐based analysis using the NCIS database provide valuable insights into the geographical distribution of deaths from TBI and the factors associated with dying outside of acute hospitals. The key finding of the present study was that 28.4% of deaths following msTBI occurred outside of acute hospital settings and approximately 1 in 6 patients died outside of any medical service area (e.g. a hospital, nursing home or other care facility). The odds of death occurring outside an acute hospital were greatest for females, patients with penetrating TBI, patients from TAS, NT, QLD, and WA, and cases where the time between injury and death was less than 1 h. The odds of death occurring outside *any* medical service area were greatest for patients aged between 18 and 64 years, injuries with a piercing object, and shorter injury‐to‐death time periods. Participants residing in SA had reduced odds of their death occurring outside any medical service area.

A previous study conducted by the ATBIND Project determined the incidence of msTBI deaths in major trauma hospitals across Australia over the same five‐year period. It was found that of the 16 350 hospital presentations for msTBI throughout the study period, approximately 14.9% died in hospital (2437 deaths in total, an average of 487 *per annum*).^9^ In this study, 2687 deaths from msTBI were recorded in hospital across the same time period. A major reason for this discrepancy is likely to be that the previous study relied on data from major trauma centres across Australia, and therefore did not include the small but clinically significant number of patients who died from msTBI at smaller hospitals (or out of hospital). In contrast, the coronial data utilised here represents all patients who died from msTBI across Australia.

From these two complementary studies conducted across the same 5‐year period, it might be estimated that of 3751 deaths from msTBI across Australia, 2437 (65.0%) people reached a hospital with the capacity to provide all options for definitive care for msTBI, and approximately 250 (6.7%) died in a non‐major trauma care facility. For 459 (12.2%) patients, death occurred in a non‐acute medical setting such as a nursing home or rehabilitation. These deaths may be due to death occurring before transfer to acute care could be sought, or because patients and families chose to not seek transfer to an acute hospital (e.g. due to advanced age of the patient). The remaining 605 (16.1%) of people died outside of any medical service. While an unknown number of these patients may have died following discharge from a hospital, it is likely that many died prior to reaching the hospital. MsTBI is associated with significant early mortality.^11^ More timely access to acute care following head injury may reduce the number of deaths occurring prior to hospital.

Overall, 28.4% of deaths attributed to msTBI occurred outside of hospital. This is lower than the figures reported by a recent Victorian study examining out‐of‐hospital and in‐hospital deaths from *all* trauma over a 10‐year period, which found that 42.6% of deaths from unintentional trauma (representing the majority of TBIs^9^) occurred outside hospital.^12^ This difference is likely because the leading cause of TBI in Australia is falls, particularly among older age groups,^9^ and the majority of trauma deaths following low falls occur in hospital.^12^ Consistent with this explanation, younger adults were found to be more likely to die outside of any medical service area compared to older people (aged 65 years and above). In contrast to older age groups who typically sustain a TBI from falls, younger age groups are more likely to sustain a msTBI from road trauma,^9^ which is associated with a significantly higher risk of dying before reaching hospital.^12^ In contrast to previous studies,^12^, ^13^ being male was associated with a lower risk of their death occurring outside of hospital from TBI, although a greater number of males sustained and died from TBI overall. The reason for this is unclear.

The strengths of the present study include the population‐based capture of causes and locations of deaths following msTBI across Australia based on ICD‐10 causes of death codes. However, it is important to acknowledge the limitations of the present study. Among deaths that occurred outside of a medical service area, it was not feasible to separately identify, with confidence, which of the following scenarios occurred: (i) death prior to the first arrival to hospital following the injury (with or without prehospital care); (ii) death following initial medical service area (acute hospital or rehabilitation) discharge but with provision for returning to acute care in the case of deterioration; and (iii) death in the setting of palliative care being provided outside a medical service area (e.g. at home). Data providing more clarity on these scenarios would enable further tailoring of system‐widen interventions for injury prevention and trauma care. There is also significant variation in reported death numbers per total population between states which may be attributed to reporting differences, possibly related to variations in which injury‐related deaths must be reported (i.e. mandatory reporting rules) in addition to state‐by‐state variations in ICD10 coding and/or the number of ICD10 codes assigned to a case. Furthermore, while cases were included in the present study if they had a medical cause of death *or* medical condition contributing to death listed as brain injury as defined by the ICD10 cause of death codes contained under S06 (intracranial injury), it may be that an included individual's “number one” cause of death was not msTBI.^9^ Similarly, the separation of primary mechanism according to whether the single data code was: “piercing, penetrating force” *versus* “blunt force” assumes that this primary mechanism was the one directly related to the TBI. Finally, the authors encourage caution in the interpretation of analyses using injury‐to‐death time data, as there will likely be cases where the time that a person died (including being found dead) may be valid but the timing of the injury event, particularly if outside a medical service area, will be an approximate estimate.

## Conclusion

A significant proportion of people with a TBI die before reaching any emergency care. Opportunities exist to improve access to emergency care for msTBI across Australia.

## Data Availability

Data sharing is not applicable to this article as no new data were created in this study. The existing data are not publicly available due to ethical restrictions.
